# 2-Amino-6-(naphthalen-1-yl)-4-phenyl­pyridine-3-carbonitrile

**DOI:** 10.1107/S1600536811002765

**Published:** 2011-03-12

**Authors:** Wei Mao, Cheng Guo, Wei Wang, Chang-jun Luan, Ren-jun Du

**Affiliations:** a Department of Applied Chemistry, College of Science, Nanjing University of Technology, Nanjing 210009, People’s Republic of China

## Abstract

In the title compound, C_22_H_15_N_3_, the naphthyl ring system makes dihedral angles of 67.40 (2) and 59.80 (3)° with the pyridyl and phenyl rings, respectively. In the crystal, the mol­ecules are connected *via* inter­molecular N—H⋯N hydrogen bonds, forming a three-dimensional network.

## Related literature

For the synthetic procedure, see: Mantri *et al.* (2008 ▶). For related structures, see: Mkhalid *et al.* (2006 ▶). For general background, see: Moreau *et al.* (1999 ▶).
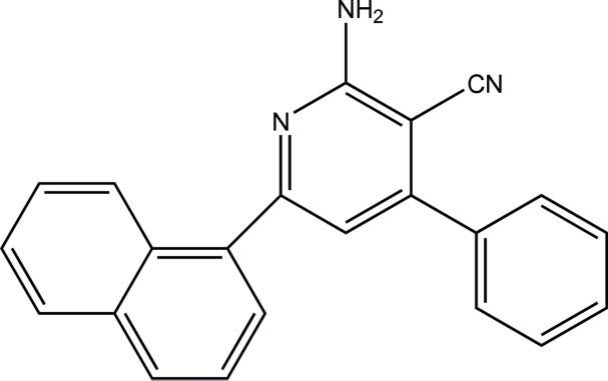


## Experimental

### 

#### Crystal data

C_22_H_15_N_3_
*M* *_r_* = 321.37
Monoclinic, 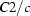

*a* = 11.799 (2) Å
*b* = 17.284 (3) Å
*c* = 17.492 (4) Å
β = 98.26 (3)°
*V* = 3530.2 (12) Å^3^
*Z* = 8
Mo *K*α radiation
μ = 0.07 mm^−1^
*T* = 293 K
0.30 × 0.20 × 0.10 mm

#### Data collection

Enraf–Nonius CAD-4 diffractometer
Absorption correction: ψ scan (North *et al.*, 1968 ▶) *T*_min_ = 0.979, *T*_max_ = 0.993
3367 measured reflections
3240 independent reflections
1717 reflections with *I* > 2σ(*I*)
*R*_int_ = 0.030
3 standard reflections every 200 reflections  intensity decay: 1%

#### Refinement

*R*[*F* ^2^ > 2σ(*F* ^2^)] = 0.066
*wR*(*F* ^2^) = 0.180
*S* = 1.01
3240 reflections
208 parameters
1 restraint
H-atom parameters constrained
Δρ_max_ = 0.13 e Å^−3^
Δρ_min_ = −0.13 e Å^−3^

### 

Data collection: *CAD-4 EXPRESS* (Enraf–Nonius, 1994) ▶; cell refinement: *CAD-4 EXPRESS* ▶; data reduction: *XCAD4* (Harms & Wocadlo,1995 ▶); program(s) used to solve structure: *SHELXS97* (Sheldrick, 2008 ▶); program(s) used to refine structure: *SHELXL97* (Sheldrick, 2008 ▶); molecular graphics: *SHELXTL* (Sheldrick, 2008 ▶); software used to prepare material for publication: *SHELXTL*.

## Supplementary Material

Crystal structure: contains datablocks I, global. DOI: 10.1107/S1600536811002765/bv2172sup1.cif

Structure factors: contains datablocks I. DOI: 10.1107/S1600536811002765/bv2172Isup2.hkl

Additional supplementary materials:  crystallographic information; 3D view; checkCIF report

## Figures and Tables

**Table 1:** Hydrogen-bond geometry (Å, °)

*D*—H⋯*A*	*D*—H	H⋯*A*	*D*⋯*A*	*D*—H⋯*A*
N2—H2*A*⋯N1^i^	0.86	2.34	3.180 (4)	165
N2—H2*B*⋯N3^ii^	0.86	2.34	3.138 (4)	154

Symmetry codes: (i) 
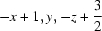
; (ii) 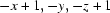.

**Fig. 1.**
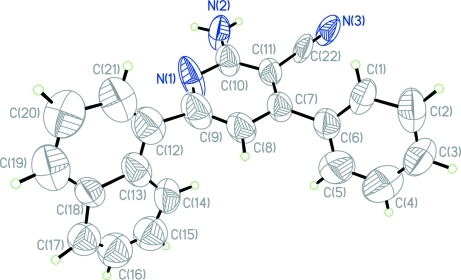
The molecular structure of (I), with the atom-numbering scheme. Displacement ellipsoids are drawn at the 50% probability level.

**Fig. 2.**
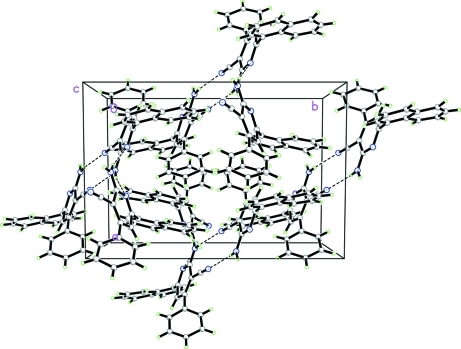
A packing diagram for (I). N—H···N hydrogen bonds are shown by dashed lines.

## supplementary crystallographic information

### Comment

The title compound, (I), contains an amino group, which can react with different groups to prepare various function organic compounds. It is a kind of aromatic organic intermediate which can be used for many fields such as medicine. (Mantri, *et al.*, 2008).  Herein we report its crystal structure. The molecular structure of (I) is shown in Fig. 1, and the selected geometric parameters are given in Table 1. The bond lengths and angles are within normal ranges. The torsion angle between the naphthyl (C12—C21) and phenyl planes (C1 to C6) is 67.40 (2)° and 59.80 (3)°, respectively. In the crystal of the title  compound, (I) was connected together *via* N—H···N intermolecular hydrogen bonds to form a three dimensional network, which seems to be very effective in the stabilization of the crystal structure.

### Experimental

The title compound, (I) was prepared by the literature method (Mantri, *et al.*, 2008). Malononitrile (20 mmol) was dissolved in EtOH (40 ml), added benzalaldehyde (20 mmol) followed by 2 drops of piperidine, the reaction mixture was refluxed for 1 h. The precipitate formed upon cooling the reaction mixture to room temperature. The crude product was filtered, and it was pure enough to carry out the further reactions. To a solution of previously synthesized benzylidene malononitrile (3 mmol, 1 equiv) in toluene was added 1-(naphthalen-1-yl)ethanone (3 mmol, 1 equiv) and ammonium acetate (4.5 mmol, 1.5 equiv). The mixture was heated in a microwave at 120° for 1 h. The reaction mixture was purified by column chromatography using dichloromethane-methanol solvent system. Crystals suitable for X-ray analysis were obtained by dissolving (I) (0.5 g) in methanol (20 ml) and evaporating the solvent slowly at room temperature for about 7 d.

### Refinement

All H atoms were positioned geometrically and constrained to ride on their parent atoms, with C—H = 0.93 Å for aromatic H and 0.86 Å for N—H, respectively. The *U*_iso_(H) = *xU*_eq_(C), where *x* = 1.2 for aromatic H, and *x* = 1.5 for other H.

### Crystal data

**Table:**

C_22_H_15_N_3_	*F*(000) = 1344
*M**_r_* = 321.37	*D*_x_ = 1.209 Mg m^−^^3^
Monoclinic, *C*2/*c*	Melting point: 438 K
Hall symbol: -C 2yc	Mo *K*α radiation, λ = 0.71073 Å
*a* = 11.799 (2) Å	Cell parameters from 25 reflections
*b* = 17.284 (3) Å	θ = 9–12°
*c* = 17.492 (4) Å	µ = 0.07 mm^−^^1^
β = 98.26 (3)°	*T* = 293 K
*V* = 3530.2 (12) Å^3^	Block, colorless
*Z* = 8	0.30 × 0.20 × 0.10 mm

### Data collection

**Table:**

Enraf–Nonius CAD-4 diffractometer	1717 reflections with *I* > 2σ(*I*)
Radiation source: fine-focus sealed tube	*R*_int_ = 0.030
graphite	θ_max_ = 25.4°, θ_min_ = 2.1°
ω/2θ scans	*h* = 0→14
Absorption correction: ψ scan (North *et al.*, 1968)	*k* = 0→20
*T*_min_ = 0.979, *T*_max_ = 0.993	*l* = −21→20
3367 measured reflections	3 standard reflections every 200 reflections
3240 independent reflections	intensity decay: 1%

### Refinement

**Table:**

Refinement on *F*^2^	Primary atom site location: structure-invariant direct methods
Least-squares matrix: full	Secondary atom site location: difference Fourier map
*R*[*F*^2^ > 2σ(*F*^2^)] = 0.066	Hydrogen site location: inferred from neighbouring sites
*wR*(*F*^2^) = 0.180	H-atom parameters constrained
*S* = 1.01	*w* = 1/[σ^2^(*F*_o_^2^) + (0.050*P*)^2^ + 3.8*P*] where *P* = (*F*_o_^2^ + 2*F*_c_^2^)/3
3240 reflections	(Δ/σ)_max_ < 0.001
208 parameters	Δρ_max_ = 0.13 e Å^−^^3^
1 restraint	Δρ_min_ = −0.13 e Å^−^^3^

### Special details

**Table:**

Geometry. All e.s.d.'s (except the e.s.d. in the dihedral angle between two l.s. planes) are estimated using the full covariance matrix. The cell e.s.d.'s are taken into account individually in the estimation of e.s.d.'s in distances, angles and torsion angles; correlations between e.s.d.'s in cell parameters are only used when they are defined by crystal symmetry. An approximate (isotropic) treatment of cell e.s.d.'s is used for estimating e.s.d.'s involving l.s. planes.
Refinement. Refinement of *F*^2^ against ALL reflections. The weighted *R*-factor *wR* and goodness of fit *S* are based on *F*^2^, conventional *R*-factors *R* are based on *F*, with *F* set to zero for negative *F*^2^. The threshold expression of *F*^2^ > σ(*F*^2^) is used only for calculating *R*-factors(gt) *etc*. and is not relevant to the choice of reflections for refinement. *R*-factors based on *F*^2^ are statistically about twice as large as those based on *F*, and *R*- factors based on ALL data will be even larger.

### Fractional atomic coordinates and isotropic or equivalent isotropic displacement parameters (Å^2^)

**Table:**

	*x*	*y*	*z*	*U*_iso_*/*U*_eq_	
N1	0.3542 (2)	0.1427 (2)	0.69970 (17)	0.1156 (13)	
C1	0.0883 (3)	0.0618 (2)	0.4510 (2)	0.1035 (13)	
H1B	0.1191	0.0169	0.4749	0.124*	
N2	0.4951 (2)	0.08860 (18)	0.64401 (14)	0.1002 (10)	
H2A	0.5373	0.0942	0.6880	0.120*	
H2B	0.5231	0.0682	0.6060	0.120*	
C2	0.0113 (4)	0.0532 (3)	0.3842 (3)	0.1361 (17)	
H2C	−0.0090	0.0049	0.3630	0.163*	
N3	0.3832 (3)	0.0285 (2)	0.45458 (14)	0.1147 (13)	
C3	−0.0341 (4)	0.1219 (4)	0.3506 (3)	0.1341 (17)	
H3A	−0.0854	0.1198	0.3050	0.161*	
C4	−0.0050 (5)	0.1915 (4)	0.3830 (3)	0.1376 (17)	
H4A	−0.0356	0.2367	0.3596	0.165*	
C5	0.0720 (4)	0.1953 (3)	0.4525 (3)	0.1277 (16)	
H5A	0.0882	0.2428	0.4765	0.153*	
C6	0.1229 (3)	0.1301 (3)	0.4846 (2)	0.0905 (11)	
C7	0.2017 (3)	0.1324 (2)	0.55800 (18)	0.0783 (9)	
C8	0.1731 (3)	0.1668 (2)	0.6244 (2)	0.0958 (12)	
H8A	0.1027	0.1917	0.6214	0.115*	
C9	0.2443 (3)	0.1661 (3)	0.6957 (2)	0.1076 (13)	
C10	0.3872 (3)	0.1114 (2)	0.63484 (18)	0.0819 (10)	
C11	0.3122 (3)	0.10289 (18)	0.56551 (16)	0.0704 (8)	
C12	0.2128 (3)	0.1874 (3)	0.7756 (3)	0.1025 (13)	
C13	0.1864 (3)	0.2620 (3)	0.7827 (2)	0.0940 (12)	
C14	0.1949 (4)	0.3212 (3)	0.7187 (2)	0.1173 (15)	
H14A	0.2117	0.3065	0.6704	0.141*	
C15	0.1767 (4)	0.3959 (3)	0.7358 (3)	0.115	
H15A	0.1869	0.4339	0.6999	0.138*	
C16	0.1480 (4)	0.4143 (3)	0.7956 (3)	0.114	
H16A	0.1390	0.4673	0.8020	0.137*	
C17	0.1279 (3)	0.3736 (2)	0.8516 (2)	0.092	
H17A	0.0958	0.3958	0.8918	0.110*	
C18	0.1548 (3)	0.2920 (2)	0.8532 (2)	0.0879 (11)	
C19	0.1452 (3)	0.2408 (3)	0.9156 (3)	0.1128 (14)	
H19A	0.1199	0.2579	0.9607	0.135*	
C20	0.1769 (4)	0.1602 (3)	0.9057 (3)	0.1198 (14)	
H20A	0.1759	0.1232	0.9442	0.144*	
C21	0.2098 (4)	0.1426 (3)	0.8311 (3)	0.1124 (14)	
H21A	0.2313	0.0916	0.8237	0.135*	
C22	0.3531 (3)	0.0621 (2)	0.50320 (16)	0.0783 (10)	

### Atomic displacement parameters (Å^2^)

**Table:**

	*U*^11^	*U*^22^	*U*^33^	*U*^12^	*U*^13^	*U*^23^
N1	0.0609 (18)	0.192 (3)	0.099 (2)	−0.014 (2)	0.0263 (16)	−0.087 (2)
C1	0.109 (3)	0.111 (3)	0.077 (2)	0.000 (2)	−0.035 (2)	0.003 (2)
N2	0.0698 (19)	0.171 (3)	0.0560 (16)	0.0290 (19)	−0.0046 (13)	−0.0224 (17)
C2	0.162 (5)	0.141 (4)	0.092 (3)	−0.015 (4)	−0.026 (3)	−0.030 (3)
N3	0.134 (3)	0.167 (3)	0.0374 (15)	0.083 (2)	−0.0051 (16)	−0.0141 (17)
C3	0.124 (4)	0.165 (5)	0.099 (3)	0.035 (4)	−0.032 (3)	−0.007 (4)
C4	0.121 (4)	0.136 (5)	0.151 (5)	0.008 (3)	0.002 (4)	0.031 (4)
C5	0.103 (3)	0.123 (4)	0.150 (4)	0.016 (3)	−0.004 (3)	−0.015 (3)
C6	0.067 (2)	0.117 (3)	0.084 (2)	0.024 (2)	0.0001 (18)	−0.036 (2)
C7	0.060 (2)	0.102 (3)	0.072 (2)	0.0134 (18)	0.0038 (16)	−0.0270 (18)
C8	0.074 (2)	0.117 (3)	0.094 (3)	0.028 (2)	0.005 (2)	−0.040 (2)
C9	0.084 (3)	0.140 (4)	0.101 (3)	0.011 (2)	0.023 (2)	−0.042 (3)
C10	0.069 (2)	0.114 (3)	0.0649 (19)	0.007 (2)	0.0169 (15)	−0.0229 (19)
C11	0.075 (2)	0.081 (2)	0.0555 (17)	0.0086 (17)	0.0104 (14)	−0.0132 (15)
C12	0.080 (3)	0.119 (4)	0.105 (3)	−0.004 (3)	0.006 (2)	−0.028 (3)
C13	0.067 (2)	0.119 (3)	0.092 (3)	0.002 (2)	−0.0036 (19)	−0.042 (3)
C14	0.112 (3)	0.154 (4)	0.077 (3)	0.022 (3)	−0.016 (2)	−0.032 (3)
C15	0.115	0.115	0.115	0.000	0.016	0.000
C16	0.114	0.114	0.114	0.000	0.016	0.000
C17	0.092	0.092	0.092	0.000	0.013	0.000
C18	0.0534 (19)	0.123 (3)	0.081 (2)	0.0281 (19)	−0.0125 (17)	−0.038 (2)
C19	0.083 (3)	0.151 (4)	0.107 (3)	−0.019 (3)	0.020 (2)	−0.023 (3)
C20	0.114 (3)	0.131 (4)	0.114 (4)	−0.032 (3)	0.016 (3)	0.014 (3)
C21	0.095 (3)	0.133 (4)	0.113 (4)	−0.024 (3)	0.026 (3)	−0.028 (3)
C22	0.082 (2)	0.120 (3)	0.0287 (14)	0.030 (2)	−0.0034 (14)	0.0008 (17)

### Geometric parameters (Å, °)

**Table:**

N1—C9	1.351 (4)	C9—C12	1.542 (5)
N1—C10	1.363 (4)	C10—C11	1.403 (4)
C1—C6	1.356 (5)	C11—C22	1.439 (4)
C1—C2	1.382 (5)	C12—C21	1.247 (5)
C1—H1B	0.9300	C12—C13	1.337 (5)
N2—C10	1.320 (4)	C13—C18	1.435 (5)
N2—H2A	0.8600	C13—C14	1.532 (6)
N2—H2B	0.8600	C14—C15	1.349 (5)
C2—C3	1.397 (6)	C14—H14A	0.9300
C2—H2C	0.9300	C15—C16	1.189 (5)
N3—C22	1.128 (4)	C15—H15A	0.9300
C3—C4	1.353 (6)	C16—C17	1.255 (5)
C3—H3A	0.9300	C16—H16A	0.9300
C4—C5	1.410 (6)	C17—C18	1.444 (5)
C4—H4A	0.9300	C17—H17A	0.9300
C5—C6	1.359 (5)	C18—C19	1.422 (5)
C5—H5A	0.9300	C19—C20	1.459 (6)
C6—C7	1.473 (5)	C19—H19A	0.9300
C7—C11	1.389 (4)	C20—C21	1.446 (6)
C7—C8	1.389 (4)	C20—H20A	0.9300
C8—C9	1.399 (5)	C21—H21A	0.9300
C8—H8A	0.9300		
			
C9—N1—C10	117.5 (3)	C7—C11—C10	120.7 (3)
C6—C1—C2	125.6 (4)	C7—C11—C22	121.4 (3)
C6—C1—H1B	117.2	C10—C11—C22	117.9 (3)
C2—C1—H1B	117.2	C21—C12—C13	119.6 (5)
C10—N2—H2A	120.0	C21—C12—C9	126.5 (5)
C10—N2—H2B	120.0	C13—C12—C9	113.9 (5)
H2A—N2—H2B	120.0	C12—C13—C18	121.4 (5)
C1—C2—C3	115.4 (4)	C12—C13—C14	122.4 (4)
C1—C2—H2C	122.3	C18—C13—C14	116.1 (4)
C3—C2—H2C	122.3	C15—C14—C13	116.6 (4)
C4—C3—C2	121.4 (4)	C15—C14—H14A	121.7
C4—C3—H3A	119.3	C13—C14—H14A	121.7
C2—C3—H3A	119.3	C16—C15—C14	121.8 (5)
C3—C4—C5	119.7 (5)	C16—C15—H15A	119.1
C3—C4—H4A	120.2	C14—C15—H15A	119.1
C5—C4—H4A	120.2	C15—C16—C17	130.1 (5)
C6—C5—C4	120.7 (5)	C15—C16—H16A	114.9
C6—C5—H5A	119.7	C17—C16—H16A	114.9
C4—C5—H5A	119.7	C16—C17—C18	119.9 (4)
C1—C6—C5	117.0 (4)	C16—C17—H17A	120.1
C1—C6—C7	121.0 (4)	C18—C17—H17A	120.1
C5—C6—C7	121.6 (4)	C19—C18—C13	119.6 (4)
C11—C7—C8	114.5 (3)	C19—C18—C17	125.5 (4)
C11—C7—C6	122.6 (3)	C13—C18—C17	114.7 (4)
C8—C7—C6	122.8 (3)	C18—C19—C20	116.9 (4)
C7—C8—C9	123.7 (3)	C18—C19—H19A	121.5
C7—C8—H8A	118.2	C20—C19—H19A	121.5
C9—C8—H8A	118.2	C21—C20—C19	114.6 (4)
N1—C9—C8	119.9 (3)	C21—C20—H20A	122.7
N1—C9—C12	112.2 (4)	C19—C20—H20A	122.7
C8—C9—C12	127.9 (3)	C12—C21—C20	127.8 (5)
N2—C10—N1	113.8 (3)	C12—C21—H21A	116.1
N2—C10—C11	123.5 (3)	C20—C21—H21A	116.1
N1—C10—C11	122.8 (3)	N3—C22—C11	178.1 (4)
			
C6—C1—C2—C3	−0.8 (7)	N1—C9—C12—C21	−64.7 (6)
C1—C2—C3—C4	−1.1 (8)	C8—C9—C12—C21	113.5 (6)
C2—C3—C4—C5	−0.5 (9)	N1—C9—C12—C13	115.7 (4)
C3—C4—C5—C6	4.2 (8)	C8—C9—C12—C13	−66.2 (6)
C2—C1—C6—C5	4.3 (7)	C21—C12—C13—C18	0.2 (6)
C2—C1—C6—C7	177.2 (4)	C9—C12—C13—C18	179.9 (3)
C4—C5—C6—C1	−5.9 (7)	C21—C12—C13—C14	176.4 (4)
C4—C5—C6—C7	−178.7 (4)	C9—C12—C13—C14	−3.9 (5)
C1—C6—C7—C11	62.2 (5)	C12—C13—C14—C15	−173.7 (4)
C5—C6—C7—C11	−125.2 (4)	C18—C13—C14—C15	2.7 (5)
C1—C6—C7—C8	−120.2 (4)	C13—C14—C15—C16	−4.9 (7)
C5—C6—C7—C8	52.3 (6)	C14—C15—C16—C17	−1.0 (8)
C11—C7—C8—C9	−5.7 (6)	C15—C16—C17—C18	8.9 (8)
C6—C7—C8—C9	176.6 (4)	C12—C13—C18—C19	−3.5 (5)
C10—N1—C9—C8	−7.9 (6)	C14—C13—C18—C19	−179.9 (3)
C10—N1—C9—C12	170.4 (4)	C12—C13—C18—C17	−179.6 (3)
C7—C8—C9—N1	11.3 (7)	C14—C13—C18—C17	4.0 (4)
C7—C8—C9—C12	−166.7 (4)	C16—C17—C18—C19	174.6 (4)
C9—N1—C10—N2	−178.7 (4)	C16—C17—C18—C13	−9.6 (5)
C9—N1—C10—C11	−0.1 (6)	C13—C18—C19—C20	4.4 (5)
C8—C7—C11—C10	−2.5 (5)	C17—C18—C19—C20	−179.9 (3)
C6—C7—C11—C10	175.2 (4)	C18—C19—C20—C21	−2.5 (5)
C8—C7—C11—C22	176.9 (3)	C13—C12—C21—C20	2.0 (7)
C6—C7—C11—C22	−5.4 (5)	C9—C12—C21—C20	−177.7 (4)
N2—C10—C11—C7	−176.0 (3)	C19—C20—C21—C12	−0.8 (7)
N1—C10—C11—C7	5.5 (6)	C7—C11—C22—N3	−68 (11)
N2—C10—C11—C22	4.7 (5)	C10—C11—C22—N3	111 (11)
N1—C10—C11—C22	−173.8 (3)		

### Hydrogen-bond geometry (Å, °)

**Table:**

*D*—H···*A*	*D*—H	H···*A*	*D*···*A*	*D*—H···*A*
N2—H2A···N1^i^	0.86	2.34	3.180 (4)	165
N2—H2B···N3^ii^	0.86	2.34	3.138 (4)	154

Symmetry codes: (i) −*x*+1, *y*, −*z*+3/2; (ii) −*x*+1, −*y*, −*z*+1.
